# Assessing the anthelmintic activity of pyrazole-5-carboxamide derivatives against *Haemonchus contortus*

**DOI:** 10.1186/s13071-017-2191-8

**Published:** 2017-05-31

**Authors:** Yaqing Jiao, Sarah Preston, Hongjian Song, Abdul Jabbar, Yuxiu Liu, Jonathan Baell, Andreas Hofmann, Dana Hutchinson, Tao Wang, Anson V. Koehler, Gillian M. Fisher, Katherine T. Andrews, Benoît Laleu, Michael J. Palmer, Jeremy N. Burrows, Timothy N. C. Wells, Qingmin Wang, Robin B. Gasser

**Affiliations:** 1 Faculty of Veterinary and Agricultural Sciences, The University of Melbourne, Parkville, VIC 3010 Australia; 2 Faculty of Science and Technology, Federation University, Ballarat, VIC 3350 Australia; 3 State Key Laboratory of Elemento-Organic Chemistry, Research Institute of Elemento-Organic Chemistry, Nankai University, Tianjin, 300071 China; 4 Medicinal Chemistry, Monash University Institute of Pharmaceutical Sciences (MIPS), Monash University, Parkville, VIC 3052 Australia; 5 Griffith Institute for Drug Discovery, Griffith University, Don Young Road, Nathan, QLD 4111 Australia; 6 Drug Discovery Biology, Monash University Institute of Pharmaceutical Sciences (MIPS), Monash University, Parkville, VIC 3052 Australia; 7 Medicines for Malaria Venture (MMV), Route de Pré-Bois 20, CH-1215 Geneva, Switzerland; 8 State Key Laboratory of Agricultural Microbiology, College of Veterinary Medicine, Huazhong Agricultural University, Wuhan, Hubei 430070 China

**Keywords:** *Haemonchus contortus*, Phenotypic screening, Tolfenpyrad, Synthetic pyrazole-5-carboxamide derivatives, Mitochondrial respiratory chain

## Abstract

**Background:**

In this study, we tested five series of pyrazole-5-carboxamide compounds (*n* = 55) for activity against parasitic stages of the nematode *Haemonchus contortus* (barber’s pole worm), one of the most pathogenic parasites of ruminants.

**Methods:**

In an optimised, whole-organism screening assay, using exsheathed third-stage (xL3) and fourth-stage (L4) larvae, we measured the inhibition of larval motility and development of *H. contortus*.

**Results:**

Amongst the 55 compounds, we identified two compounds (designated **a-15** and **a-17**) that reproducibly inhibit xL3 motility as well as L4 motility and development, with IC_50_ values ranging between ~3.4 and 55.6 μM. We studied the effect of these two ‘hit’ compounds on mitochondrial function by measuring oxygen consumption. This assessment showed that xL3s exposed to each of these compounds consumed significantly less oxygen and had less mitochondrial activity than untreated xL3s, which was consistent with specific inhibition of complex I of the respiratory electron transport chain in arthropods.

**Conclusions:**

The present findings provide a sound basis for future work, aimed at identifying the targets of compounds **a-15** and **a-17** and establishing the modes of action of these chemicals in *H. contortus*.

**Electronic supplementary material:**

The online version of this article (doi:10.1186/s13071-017-2191-8) contains supplementary material, which is available to authorized users.

## Background

Synthetic pyrazole-5-carboxamide derivatives, such as tebufenpyrad and tolfenpyrad, are important pesticides which are recognized to inhibit complex I of the mitochondrial electron transport (respiratory) chain [1, 2]. Tebufenpyrad, which was first discovered in 1987 by Mitsubishi Kasei Co., Ltd., has known activity against selected Homoptera and phytophagous mites [3]. Tolfenpyrad was discovered by Mitsubishi Chemical Corporation (now Nihon Nohyaku Co., Ltd.) and developed for the control of various agricultural pests, including Acarina, Coleoptera, Diptera, Hemiptera, Lepidoptera and Thysanoptera (Arthropoda); the latter chemical is active mainly upon contact with egg, larval, nymphal and/or adult stages [4]. Because of the effectiveness of these two pyrazole-5-carboxamides in controlling such agricultural pests [4, 5], there has been a considerable commercial interest in synthesizing various structural derivatives, with changes being made to the pyrazole and/or benzene rings [5–12], but little work has been done to alter the chemical bridge between the pyrazole and benzene rings (cf. [2]).

Although tebufenpyrad, tolfenpyrad and selected derivatives [2] have been developed to kill arthropod pests, we recently showed, in a compound screen of the ‘Pathogen Box’ (www.pathogenbox.org) from the Medicines for Malaria Ventures (MMV; www.mmv.org), that the latter compound has an exquisite in vitro activity against parasitic stages of the barber’s pole worm, *Haemonchus contortus* (Nematoda: Strongylida) [13]. Indeed, tolfenpyrad reproducibly and irreversibly inhibits the motility of exsheathed third-stage (xL3s) and fourth-stage larvae (L4s) of this parasitic nematode, and also the growth and development of L4s, with IC_50_ values ranging between 0.03 and 3.1 μM after 72 h of exposure. We demonstrated that xL3s exposed to tolfenpyrad consumed significantly less oxygen than unexposed xL3s, which was consistent with a specific inhibition of complex I of the respiratory electron transport chain in the mitochondrion (cf. [1]). In vitro cytotoxicity data indicated that tolfenpyrad is ≥ 18-fold more selective for *H. contortus* than a mammalian cell line [13], raising the possibility of repurposing this agent against (at least some) parasitic nematodes and/or hit-to-lead optimisation.

In general terms, this evidence suggested that some pyrazole-5-carboxamide compounds developed as agricultural pesticides (against arthropods of plants) might also be able to be repurposed to other ecdysozoans, such as nematodes, provided that they are sufficiently safe for application/administration to animals and/or the environment. Therefore, we sourced two published series of pyrazole-5-carboxamides [2, 14] and three new series of new pyrazole-5-carboxamides analogues (Song et al., unpublished). The availability of this small library provided us with an opportunity to logically extend our recent study [13]. Here, we evaluated the activity of these pyrazole-5-carboxamides derivatives (*n* = 55) against parasitic stages of the nematode *Haemonchus contortus* and compared their potency with the two original, commercially available chemicals, tebufenpyrad and tolfenpyrad. We used this whole-organism screening assay to measure the inhibition of larval motility and development of *H. contortus*, and then investigated the effect of any active compound on mitochondrial function by measuring oxygen consumption in this nematode.

## Methods

### Procurement of *H. contortus*

The Haecon-5 strain of *H. contortus* was maintained in experimental sheep as described previously [15] and in accordance with institutional animal ethics guidelines (permit no. 1413429; The University of Melbourne, Australia). L3s were produced from *H. contortus* eggs by incubating humidified faeces from infected sheep at 27 °C for 1 week [15], sieved through nylon mesh (pore size: 20 μm) to remove debris or dead larvae and then stored at 10 °C for a maximum of 3 months. For screening and basal oxygen consumption measurements (see following Sub-sections), L3s were exsheathed and sterilised in 0.15% v/v sodium hypochlorite (NaClO) at 37 °C for 20 min [15]. Thereafter, xL3s were washed five times in sterile physiological saline by centrifugation at 600× *g* (5 min) at 22–24 °C. Then, xL3s were immediately suspended in Luria Bertani medium [LB: 10 g of tryptone (cat no. LP0042; Oxoid, Hampshire, England), 5 g of yeast extract (cat no. LP0042; Oxoid) and 5 g of NaCl (cat. no. K43208004210; Merck, Kenilworth, NJ, USA)] in 1 l of reverse-osmosis deionised water). LB was autoclaved and supplemented with 100 IU/ml of penicillin, 100 μg/ml of streptomycin and 2.5 μg/ml of amphotericin (Fungizone, antibiotic - antimycotic; cat. no. 15240-062; Carlsbad, CA, USA); this supplemented LB was designated LB*.

### Pyrazole-5-carboxamide compounds

A total of 55 analogs of tebufenpyrad and tolfenpyrad were derivatised (Additional file 1: Table S1). In brief, pyrazole derivatives containing α-hydroxymethyl-N-benzyl carboxamide, α-chloromethyl-N-benzyl carboxamide and 4,5-dihydrooxazole moieties (**a-1** to **a-23**) [2], carbohydrazide (**b-1** to **b-7**), imine, oxime ether, oxime ester, and dihydroisoxazoline (**c-1** to **c-15**) [14], oxazole (**d-1** to **d-8**) and **e-1** to **e-2** (this study) were designed and synthesized (Additional file 1: Table S1). In addition, tolfenpyrad (IUPAC name: 4-chloro-N-[[4-(1,1-dimethylethyl)phenyl]methyl]-3-ethyl-1-methyl-1H-pyrazole-5-carboxamide, cat. no. T535325, Toronto Research Chemicals, Toronto, Ontario, Canada) and tebufenpyrad (IUPAC name: 4-chloro-3-ethyl-1-methyl-N-[4-(p-tolyloxy)benzyl]pyrazole-5-carboxamide, cat. no. T013500, Toronto Research Chemicals, Canada) (99.9% purity) were purchased from commercial suppliers; the former chemical was used as a positive-reference compound (cf. [13]).

### Screening of chemicals, inhibitory concentrations and cytotoxicity assessment

All compounds (Additional file 1: Table S1) were prepared as described previously [13] and screened (in triplicate) at a concentration of 100 μM on xL3s of *H. contortus* in 96-well microculture plates using two assay-control compounds, monepantel (Zolvix, Novartis Animal Health, Basel, Switzerland) and moxidectin (Cydectin, Virbac, France). In brief, compounds were dissolved to a stock concentration of 10 mM in dimethyl sulfoxide (DMSO, Ajax Finechem, Melbourne, Australia), individually diluted to the final concentration of 100 μM using LB*, and dispensed (in triplicate) into wells of the 96-well microculture plates (cat no. 3635; Corning Life Sciences, Corning, NY, USA) using a multichannel pipette. In addition, negative-controls (LB* and LB* + 0.5% DMSO; six wells each) and positive-controls (final concentration of 100 μM of monepantel and 100 μM of moxidectin; triplicate wells each) were included. Then, xL3s (~300/well) were dispensed into wells of the plate. Following an incubation for 72 h at 38 °C and 10% CO_2_, a video recording (5 s) was taken of each well of the 96-well microculture plate (containing xL3s) using a grey-scale camera (Rolera bolt, Q imaging Scientific Coms, Canada), and a motorised X-Y axis stage (BioPoint 2, Ludl Electronics Products, Hawthorne, NY, USA). Individual video recordings were processed to calculate a motility index (MI) using an algorithm described previously [13, 15]. MIs were normalised to the positive- and negative-controls (to remove plate-to-plate variation) using the program Prism (v.6 GraphPad Software, USA). A compound was recorded as having activity if it reduced xL3 motility by ≥ 70% after 72 h of incubation.

Anti-xL3 activity of individual compounds was confirmed, and half maximum inhibitory concentration (IC_50_) values estimated from dose-response curves (24 h, 48 h and 72 h). Compounds that reduced the motility of xL3s were also tested for their ability to inhibit the development of xL3s to L4s, and the motility of L4s. All assays (for xL3 motility, and L4 development and motility) were performed in triplicate, between 3-5 times on different days. To determine IC_50_ values, the data from each assay (xL3 motility, L4 motility and development) were converted to a percentage with reference to the negative-control (LB* + 0.5% DMSO), and IC_50_ values determined using a variable slope four-parameter equation, constraining the top value to 100% and using a least squares (ordinary) fit model (v.6 GraphPad Software). The toxicity of selected compounds was measured by assessing their inhibition of the proliferation of human neonatal foreskin fibroblast (NFF) cells as described previously [16]. Selectivity indices (SIs) were calculated as follows: IC_50_ for NFF cells / IC_50_ for *H. contortus*).

### Oxygen consumption assay

Oxygen consumption (reflecting oxidative phosphorylation) was measured in the medium containing *H. contortus* xL3s (*n* = 600 per well) in the presence (50 μM or 100 μM) or absence of individual chemical compounds using the Seahorse XFe96 analyzer (Seahorse Biosciences, Agilent Technologies, Santa Clara, CA, USA) as described previously with minor modification [13]. In brief, xL3s were dispensed into XFe96 cell culture microplates (Seahorse Biosciences, Aligent Technologies) at a density of 600 xL3s per well in 150 μl of XF Base medium (Seahorse Bioscience, USA), supplemented with 4.5 g/l of glucose, 0.5 mM of sodium pyruvate and 2 mM of glutamine (Sigma-Adlrich, St. Louis, MI, USA) (pH = 7.4). Four wells contained XF Base medium alone and served as normalisation controls. Subsequently, compounds dissolved in 25 μl of XF Base medium were individually loaded into the injection ports (in quadruplicate), and programmed to dispense into the XFe96 microplate after 5 measurements of respiration at 6 min intervals (2 min-mix; 4 min-measure). Using this approach, xL3s were exposed to compound concentrations of 100 μM, 50 μM and 0 μM. Respiration rates were measured every 6 min for a period of 180 min. Assays were repeated three times on separate days.

## Results

**Fig. 1:**
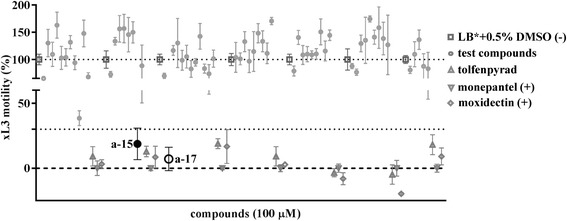
Primary screening of compounds on *Haemonchus contortus*. Synthetic pyrazole-5-carboxamides (*n* = 55) were tested for inhibition of motility of exsheathed third-stage larvae (xL3) of *H. contortus* at a concentration of 100 μM (after 72 h of exposure). Tolfenpyrad, monepantel and/or moxidectin were also included as control compounds; tolfenpyrad was used as the positive reference-control, as it has known activity against xL3s and L4s of *H. contortus* [13]. Two active compounds, **a-15** and **a-17**, inhibited xL3 motility by ≥ 70%. Supplementary file 2 shows the “straight” phenotype in larvae exposed to **a-15** and **a-17**, similar to that of the tolfenpyrad control

**Fig. 2:**
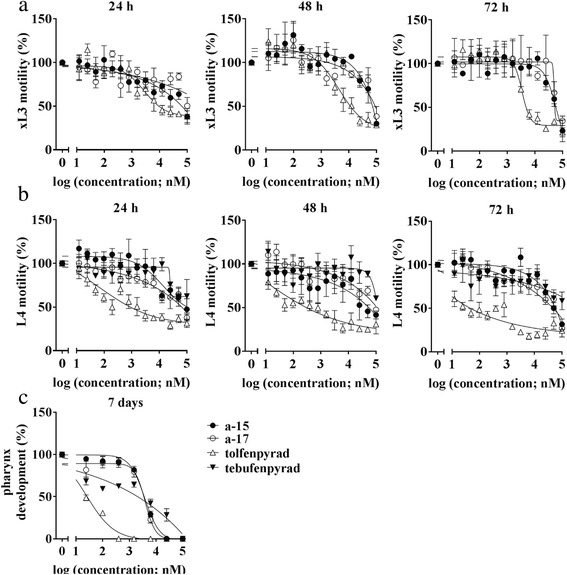
Dose-response curves. The effects of the active pyrazole-5-carboxamide test compounds, **a-15** and **a-17**, tolfenpyrad and tebufenpyrad on parasitic stages of *Haemonchus contortus* in vitro. Inhibition of the motility of third-stage larvae (xL3s) at 24 h, 48 h and 72 h (**a**) for individual compounds motility (**b**) and inhibition of development (**c**) of fourth-stage larvae (L4s) after seven days. Each data point represents the mean of three experiments (± standard error of the mean, SEM)

In the primary screen of the 55 test compounds (Fig. 1), two pyrazole-5-carboxamide derivatives, **a-15** and **a-17**, were recorded to inhibit xL3 motility by ≥ 70% at concentrations of 50 μM or 100 μM, both revealing a straight phenotype (Fig. 1; Additional file 2). Subsequent assays using xL3s of *H. contortus* showed that the potency of these two test compounds at 72 h of incubation (IC_50_ values = 55.63 ± 0.18 μM and 51.60 ± 1.41 μM, respectively) was considerably less than tolfenpyrad (IC_50_ value = 3.05 ± 0.47 μM) (Fig. 2; Table 1).

**Table 1:** In vitro activity of test compounds. Comparison of activity of compounds **a-15** and **a-17** with tolfenpyrad and tebufenpyrad on the motility of exsheathed third-stage (xL3) and fourth-stage (L4) larvae of *Haemonchus contortus* (after 24 h, 48 h and 72 h of exposure) and on the development of L4 (after 7 days of exposure)

Compound	Half maximum inhibitory concentration (IC_50_ μM)						
	xL3 motility			L4 motility			L4 development
	24 h	48 h	72 h	24 h	48 h	72 h	7 days
**a-15**	–	–	55.63 ± 0.18	–	–	26.31^a^	3.97 ± 0.35
**a-17**	–	–	51.60 ± 1.41	–	–	15.58^a^	3.42 ± 0.50
Tolfenpyrad	2.98 ± 0.50	3.85 ± 1.33	3.05 ± 0.47	0.12 ± 0.07	0.06 ± 0.02	0.03 ± 0.02	0.08 ± 0.01
Tebufenpyrad	na	na	na	> 100	> 100	> 100	6.70^a^

^a^Half maximum inhibitory concentration could not be accurately calculated by the log (agonist) versus response - variable slope (four parameter) equation, a IC_50_ value was estimated

*na* no activity; “–” indicates where IC_50_ values were not determined if the log(agonist) *vs* response - variable slope (four parameters) model could not be used to fit the curve

**Fig. 3:**
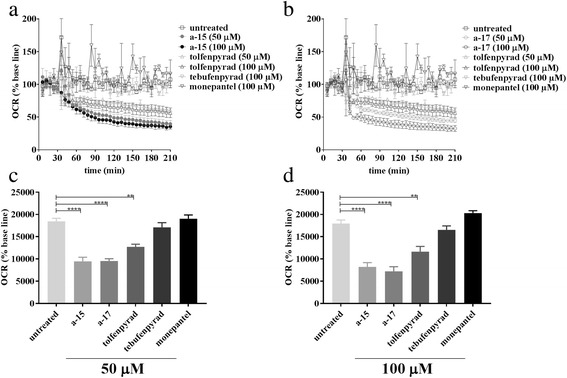
Respiration rates of *Haemonchus contortus* treated with test or control compounds in vitro. Panels **a** and **b** show individual curves of the oxygen consumption rate (OCR) of third larvae (xL3s) (*n* = 600 per well) following exposure to individual test compounds (**a-15** and **a-17**) and reference controls (tolfenpyrad, tebufenpyrad and monepantel), tested at concentrations of 50 μM and 100 μM, respectively. The OCR data were measured 35 times (2 min-mix 4 min-measure) for 30 min before and 180 min after exposure to each compound using a Seahorse XFe96 flux analyser. Three separate experiments were conducted using 4 replicates in each experiment. Panels **c** and **d** show the total oxygen consumption of xL3s (*n* = 600 per well; calculated from the area under the curve, AUC) following exposure to individual test compounds (**a-15** and **a-17**) and the reference control compounds (tolfenpyrad, tebufenpyrad and monepantel), tested at concentrations of 50 μM and 100 μM, respectively. Variation was expressed as the standard error of the mean (SEM). Significance between values (mean ± SEM) was determined using a nonparametric (Kruskal-Wallis) one-way ANOVA and Dunnett’s multiple comparison test. *Asterisks* indicate values that are significantly different from one another (**P < 0.01; ****P < 0.0001)

In the L4 motility assay, test compounds **a-15**, **a-17** and tolfenpyrad all had significant inhibitory activities, but IC_50_ values of the test compounds (~15–26 μM) at 24 h, 48 h and 72 h were considerably greater than those of the reference control (tolfenpyrad; 0.03 ± 0.02 μM) at the 72 h time point (Table 1). In the L4 development assay (at 7 days), **a-15** (IC_50_ of 3.97 ± 0.35 μM) and **a-17** (IC_50_ of 3.42 ± 0.50 μM) had significantly less inhibitory effect on the L4 development than did tolfenpyrad (IC_50_of 0.08 ± 0.01 μM; one-way ANOVA (*F*_(2,6)_ = 35.52, *P* = 0.0005) and Dunnett’s multiple comparison test (*P =* 0.0004 for **a-15**, and *P* = 0.001 for **a-17**)). Interestingly, while tolfenpyrad had the expected inhibitory effect on the motility of xL3 and L4 stages (cf. [13]), tebufenpyrad reduced the motility and development of L4s, but not of xL3s. Using available cytotoxicity information (cf. Table 2), selective indices (SIs) of **a-15** (1.2, 2.5 and 16.8), **a-17** (i.e. 1.4, 4.6 and 21.1) and tolfenpyrad (i.e. > 16, 1667 and 625) for xL3 motility, L4 motility and L4 development, respectively, were relatively high for the L4s. Subsequently, it was assessed whether **a-15** and **a-17** would inhibit mitochondrial respiration in xL3s of *H. contortus* by measuring oxygen consumption over time (Fig. 3). The results showed that **a-15, a-17-** and tolfenpyrad-treated xL3s consumed significantly less oxygen than untreated xL3s at 50 μM and 100 μM (one-way ANOVA (*F*_(5,12)_ = 30.18, *P <* 0.0001 at 50 μM; *F*_(5,12)_ *=* 34.57, *P* < 0.0001 at 100 μM) and Dunnett’s multiple comparison test (*P* = 0.0001, 0.0001 and 0.0012 for **a-15**, **a-17** and tolfenpyrad at 50 μM, respectively; *P =* 0.0001, 0.0001 and 0.0016 for **a-15**, **a-17** and tolfenpyrad at 100 μM, respectively) (see Fig. 3). As expected, based on its distinct mode of action (cf. [17]), monepantel resulted in an inhibition of larval motility and development, but it did not significantly reduce oxygen consumption at the time-point measured. Similarly, tebufenpyrad did not inhibit respiration.

**Table 2:** Toxicity assessment. Compounds **a-15** and **a-17** were tested for toxic effects on a human neonatal foreskin fibroblast (NFF) cell line; tolfenpyrad was included as a reference control. Selectivity indices of these compounds on the motility of exsheathed third- and fourth-stage larvae (at 72 h) (xL3s and L4s) and the development of L4s (at 7 days) were calculated using a recognised formula [16]

Compound	IC_50_ (μM) for NFF cells	Selectivity index (SI) for *H. contortus*		
		xL3 motility	L4 motility	L4 development
**a-15**	66.72 ± 10.04	1.20	2.54	16.81
**a-17**	72.15 ± 1.61	1.40	4.63	21.10
Tolfenpyrad	> 50	> 16.40	> 1666.70	> 625

## Discussion

The screening of 55 novel pyrazole-5-carboxamide derivatives identified two compounds (designated **a-15** and **a-17**) with major activity against parasitic larval stages (xL3 and L4) of *H. contortus* in vitro; IC_50_ values were compared with tolfenpyrad, a pyrazole-5-carboxamide insecticide, which was recently shown, for the first time, to have substantial anthelmintic activity [13].

In the present study, compounds **a-15** and **a-17**, like tolfenpyrad, were shown to be more potent against L4s than xL3s based on IC_50_ values (larval motility and development). It is not known whether the potency difference of each of these two compounds between xL3s and L4s is due to variation in drug uptake, metabolism and/or mode of action, but it is likely that uptake of the chemicals is considerably greater in the L4 stage, as it has a functional pharynx and has a substantial nutrient requirement at this stage of development [18]. It may also be that the target(s) of these chemicals is/are expressed at a higher level in L4s than xL3s, achieving greater binding and inhibitory effects in the nematode. Although compounds **a-15** and **a-17** have less anthelmintic effect than tolfenpyrad on *H. contortus* (Table 1), these compounds, which contain α-hydroxymethyl-N-benzyl carboxamide, still have considerable anthelmintic activity and appear to have a reasonable level of selectivity. A comparison with results from a previous study [2] shows that both of the test compounds with anti-*H. contortus* effect(s) also have considerable activity against some plant-parasitic insects. This finding is in accord with that of tolfenpyrad, which is reported to have a relatively broad spectrum of activity against some arthropods of plants [19] and selected nematode species (ref. [13] and Preston et al., unpublished data).

Published information has implied that the mode of action of some pyrazole derivatives, such as tolfenpyrad (fungicide or insecticide/acaricide), also relates to a specific inhibition of complex I in the respiratory electron transport chain in mitochondria (e.g. [14, 20]. Therefore, to provide support for the hypothesis that compounds **a-15** and **a-17** act to disrupt or interrupt mitochondrial function, resulting in a loss of parasite motility and viability, oxygen consumption (via oxidative phosphorylation) was measured in real-time in xL3s of *H. contortus* using oxygen-sensitive probes (cf. [21, 22]). Both compounds were shown (within 180 min) to reduce, in a dose-dependent manner, oxygen consumption in *H. contortus*. This reduction in consumption preceded the inhibition of motility and development in this nematode, indicating that each of the two compounds significantly decreases oxidative phosphorylation, resulting (directly and/or indirectly) in a substantial inhibition of larval motility and development. The fact that these two compounds induce a similar phenotype to that caused by tolfenpyrad [13] calls for future studies to confirm that the electron transport chain is indeed the target. Such future studies might be conducted in the free-living nematode *C. elegans* (which is related to *H. contortus*), because it is very amenable to gene knockdown or knockout experiments, in contrast to *H. contortus* [23–25]. In addition, other investigations could be conducted in *C. elegans* to establish whether any other pathways or (e.g. stress) responses are involved in enabling or exacerbating the anthelmintic effects of these compounds.

Interestingly, a previous study [26] had identified nafuredin as a nematocide against *H. contortus* in vivo in sheep; this chemical selectively targets mitochondrial complex I and disrupts the anaerobic NADH-fumarate reductase respiratory pathway in helminths [26]. Moreover, nafuredin has been shown to inhibit mitochondrial complex I of *Ascaris* [27], and is more than 1000-fold selective on this nematode than rat liver cells [26, 28]. However, the original drug was not of practical use or commercialised as a nematocide, because it had been shown chemically unstable in air due to the presence of oxygen-labile conjugated dienes (cf. [28]). Adapting to the developmental and environmental alterations, parasitic worms use mitochondria complex I in respiratory chain, with rhodoquinone and ubiquinone as electron receptors in parasitic stages (anaerobic) and free-living larvae stages (aerobic), respectively. Notably, mammalian complex I of nematodes differs considerably from complex I in mammals mitochondria [28–31]. Thus, based on evidence from the present and previous studies, it appears that elements of mitochondrial respiratory chain in parasitic worms seem to have promising potential as targets for nematocides.

## Conclusions

The present findings provide a sound basis for future work, aimed at identifying the targets of compounds **a-15**, **a-17** and tolfenpyrad, and establishing the modes of action of these chemicals in *H. contortus*. In addition, medicinal chemistry-based structure activity relationship (SAR) studies of pyrazole-5-carboxamides will enhance understanding of which features of the pyrazole-5-carboxamides skeleton are vital for anthelmintic activity.

## Additional files

Additional file 1: Table S1. The 55 pyrazole-5-carboxamide derivatives synthesized *de novo.* (DOCX 900 kb)

Additional file 2: Five-second video recordings of *Haemonchus contortus* exsheathed third-stage larvae (xL3s) showing the “straight” phenotype induced by exposure to the pyrazole-5-carboxamides **a-15**, **a-17 (**test compounds) or tolfenpyrad (100 μM). Videos of xL3s exposed to the same concentration of tebufenpyrad, monepantel or moxidectin, or not exposed to any compound (LB* + 0.5% DMSO) were also included for comparison. (ZIP 148083 kb)

## Abbreviations

IC_50_: Half maximum inhibitory concentration values
L4: fourth-stage larvae
LB: Luria Bertani medium
MI: Motility index
MMV: Medicines for Malaria Ventures
NFF cells: Human neonatal foreskin fibroblast cells
SAR: Structure activity relationship
Sis: Selectivity indices
xL3: exsheathed third-stage larvae
