# Supplementary material for: Assessing the anthelmintic activity of pyrazole-5-carboxamide derivatives against Haemonchus contortus
Source: Parasit Vectors. 2017 May 31;10:272. doi: 10.1186/s13071-017-2191-8 (PMC5452367; doi:10.1186/s13071-017-2191-8)
Supplement: Supplementary file 1 — The 55 pyrazole-5-carboxamide derivatives synthesized de novo. (DOCX 900 kb) [file 13071_2017_2191_MOESM1_ESM.docx]

**Additional file 1: Table S1** Features of tolfenpyrad and tebufenpyrad as well as 55 *de novo*-synthesized pyrazole-5-carboxamide derivatives (series a to e) tested in this study.

| Compound | Structure | Formula and Molecular Weight |
| --- | --- | --- |
| **Tolfenpyrad** |  | Chemical Formula: C_21_H_22_ClN_3_O_2_  Molecular Weight: 383.8713 |
| **Tebufenpyrad** |  | Chemical Formula: C_18_H_24_ClN_3_O  Molecular Weight: 333.8557 |
| **a-1** |  |  |
| **a-2** |  |  |
| **a-3** |  |  |
| **a-4** |  |  |
| **a-5** |  |  |
| **a-6** |  |  |
| **a-7** |  |  |
| **a-8** |  |  |
| **a-9** |  |  |
| **a-10** |  |  |
| **a-11** |  |  |
| **a-12** |  |  |
| **a-13** |  |  |
| **a-14** |  |  |
| **a-15** |  |  |
| **a-16** |  |  |
| **a-17** |  |  |
| **a-18** |  |  |
| **a-19** |  |  |
| **a-20** |  |  |
| **a-21** |  |  |
| **a-22** |  |  |
| **a-23** |  |  |
| **b-1** |  |  |
| **b-2** |  |  |
| **b-3** |  |  |
| **b-4** |  |  |
| **b-5** |  |  |
| **b-6** |  |  |
| **b-7** |  |  |
| **c-1** |  |  |
| **c-2** |  |  |
| **c-3** |  |  |
| **c-4** |  |  |
| **c-5** |  |  |
| **c-6** |  |  |
| **c-7** |  |  |
| **c-8** |  |  |
| **c-9** |  |  |
| **c-10** |  |  |
| **c-11** |  |  |
| **c-12** |  |  |
| **c-13** |  |  |
| **c-14** |  |  |
| **c-15** |  |  |
| **d-1** |  |  |
| **d-2** |  |  |
| **d-3** |  |  |
| **d-4** |  |  |
| **d-5** |  |  |
| **d-6** |  |  |
| **d-7** |  |  |
| **d-8** |  |  |
| **e-1** |  |  |
| **e-2** |  |  |
